# Aerobic exercise training rescues cardiac protein quality control and blunts endoplasmic reticulum stress in heart failure rats

**DOI:** 10.1111/jcmm.12894

**Published:** 2016-06-16

**Authors:** Luiz H. M. Bozi, Paulo R. Jannig, Natale Rolim, Vanessa A. Voltarelli, Paulo M. M. Dourado, Ulrik Wisløff, Patricia C. Brum

**Affiliations:** ^1^ School of Physical Education and SportUniversity of Sao PauloSao PauloBrazil; ^2^ Institute of Biomedical SciencesUniversity of Sao PauloSao PauloBrazil; ^3^ K.G. Jebsen Center of Exercise in Medicine at Department of Circulation and Medical ImagingNorwegian University of Science and Technology ‐ NTNUTrondheimNorway; ^4^ Department of PharmacologyInstitute of Biomedical ScienceUniversity of Sao PauloSao PauloBrazil

**Keywords:** exercise, myocardial infarction, endoplasmic reticulum stress, protein quality control

## Abstract

Cardiac endoplasmic reticulum (ER) stress through accumulation of misfolded proteins plays a pivotal role in cardiovascular diseases. In an attempt to reestablish ER homoeostasis, the unfolded protein response (UPR) is activated. However, if ER stress persists, sustained UPR activation leads to apoptosis. There is no available therapy for ER stress relief. Considering that aerobic exercise training (AET) attenuates oxidative stress, mitochondrial dysfunction and calcium imbalance, it may be a potential strategy to reestablish cardiac ER homoeostasis. We test the hypothesis that AET would attenuate impaired cardiac ER stress after myocardial infarction (MI). Wistar rats underwent to either MI or sham surgeries. Four weeks later, rats underwent to 8 weeks of moderate‐intensity AET. Myocardial infarction rats displayed cardiac dysfunction and lung oedema, suggesting heart failure. Cardiac dysfunction in MI rats was paralleled by increased protein levels of UPR markers (GRP78, DERLIN‐1 and CHOP), accumulation of misfolded and polyubiquitinated proteins, and reduced chymotrypsin‐like proteasome activity. These results suggest an impaired cardiac protein quality control. Aerobic exercise training improved exercise capacity and cardiac function of MI animals. Interestingly, AET blunted MI‐induced ER stress by reducing protein levels of UPR markers, and accumulation of both misfolded and polyubiquinated proteins, which was associated with restored proteasome activity. Taken together, our study provide evidence for AET attenuation of ER stress through the reestablishment of cardiac protein quality control, which contributes to better cardiac function in post‐MI heart failure rats. These results reinforce the importance of AET as primary non‐pharmacological therapy to cardiovascular disease.

## Introduction

Cardiovascular diseases are the main cause of morbidity and mortality worldwide 1. The mechanisms involved in pathogenesis and progression of cardiovascular diseases include renin angiotensin and sympathetic systems hyperactivation, reduced mitochondrial function and Adenosine triphosphate (ATP) levels, calcium imbalance and increased reactive oxygen species production in hearts of patients and animal models of cardiovascular diseases 2, 3, 4, 5. Such maladaptations disrupt the proper protein folding leading to accumulation of misfolded proteins in endoplasmic reticulum (ER), which intensifies cardiomyocyte and heart dysfunctions 6, 7. This condition referred as ER stress, triggers the unfolded protein response (UPR). Initially, UPR inhibits protein synthesis and increases both ER protein folding and degradation capacities, making an effort to reestablish the ER homoeostasis 6. However, if the ER stress persists, prolonged UPR activation leads to apoptotic cell death 6. Although progression towards apoptotic phase of UPR seems to contribute to onset of heart failure, there are no pharmacological or non‐pharmacological strategies available to counteract cardiac ER stress.

Aerobic exercise training (AET) is an efficient adjuvant therapy for prevention and treatment of a variety of cardiovascular diseases, since it improves exercise capacity, cardiac function and attenuates cardiac remodelling 8, 9, 10. In the last years, our lab has demonstrated in heart failure animal models that AET promotes a broad range of cardiac beneficial effects, such as attenuation of calcium imbalance, renin angiotensin and sympathetic systems activation, mitochondrial dysfunction and oxidative stress 2, 3, 11, being a potential therapeutic tool to attenuate ER stress. In fact, AET attenuates ER stress in hepatic and adipose tissues of obese rats 12. However, no information is provided about the impact of AET on ER stress in cardiovascular diseases.

In this study, we tested whether AET would reduce cardiac ER stress and UPR activation leading to improved protein quality control and cardiac function in post‐myocardial infarction (MI) heart failure rats.

## Materials and methods

Detailed methods are available in Data S1.

## Results

### Improved cardiac function by AET in post‐MI heart failure rats is associated with attenuation of cardiac ER stress

To verify whether AET would blunt MI‐induced ER stress and reestablish cardiac contractility, we evaluated protein levels of key ER stress markers, cardiac function and exercise tolerance in untrained and trained MI rats. Sham‐operated rats were considered as control healthy group.

**Figure 1:**
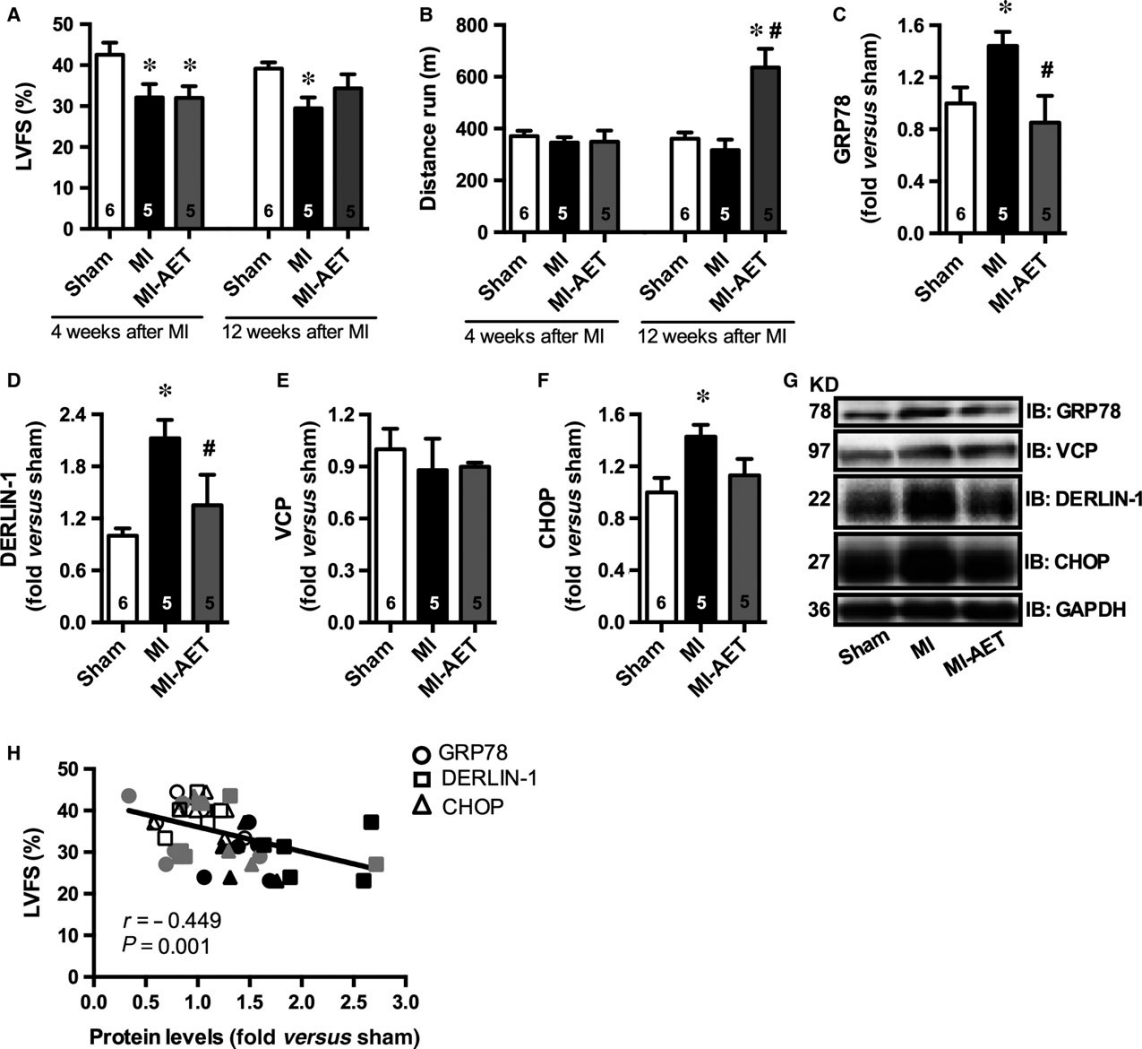
Cardiac function, exercise capacity and protein levels of endoplasmic reticulum stress markers in sham (white), MI (black) and MI under AET (grey). Left ventricle fractional shortening at 4th and 12th weeks after MI induction (LVFS) (**A**). Distance run at 4th and 12th weeks after MI induction (**B**). Protein levels of GRP78 (**C**), DERLIN‐1 (**D**), VCP (**E**) and CHOP (**F**). Representative immunoblots (**G**), and correlation between protein levels of ER stress makers (GRP78, DERLIN‐1 and CHOP) and LVFS (**H**). Data presented as mean ± S.E.M. Sham refers to rats submitted to fictitious surgery, MI refers to myocardial infarction and AET refers to aerobic exercise training. * indicates *P* ≤ 0.05 versus sham, ^#^ indicates *P* ≤ 0.05 versus MI. The number of animals in each analysis is shown within the bars.

Myocardial infarction rats (12 weeks after MI surgery) showed symptoms of heart failure, such as lung oedema, LV remodelling and dysfunction (Fig. 1A and Table S1 and S2) despite no differences were observed in exercise capacity between Sham and MI sedentary animals (Fig. 1B). The LV dysfunction observed in MI rats was paralleled by increased ER protein levels of glucose‐regulated protein 78 (GRP78), DERLIN‐1 and C/EBP homologous protein (CHOP) (Fig. 1C, D, F, and G). However, no differences were observed in Valosin‐containing protein (VCP) protein levels (Fig. 1E and G) among groups. Together, these data demonstrate that MI leads to heart failure and ER stress.

Eight weeks of AET (from 4th to 12th weeks after MI) increased exercise capacity and attenuated LV dysfunction (Fig. 1A and B) with no impact on lung oedema, heart‐to‐bw ratio and MI extension (Table S1). Indeed, AET blunted ER stress by attenuating protein levels of GRP78, CHOP and DERLIN‐1 (Fig. 1C, D, F and G). Of interest, a significant negative correlation between protein levels of ER stress markers and LV fractional shortening (Fig. 1H) further suggests that AET‐induced improved cardiac function was related to attenuated ER stress in MI rats. The impact of AET on ER stress markers was specific for MI exercised group, since AET in Sham animals displayed no effects on GRP78, VCP and CHOP protein levels with a slight increase in DERLIN‐1 (13%) mainly because of ER protein turnorver induced by a AET (Fig. S1). These findings corroborate previous findings that AET failed to induce any changes on cardiac protein levels of ER stress markers in healthy rodents 13.

### AET‐induced attenuation of cardiac ER stress is related to reestablishment of cardiac protein quality control

As we observed increased levels of DERLIN‐1 in MI rats, a protein involved in ER‐associated protein degradation, we further investigated whether cardiac protein quality control would be impaired in MI rats and returned to sham levels by AET. To test this hypothesis, we evaluated several steps involved in cardiac protein quality control, such as misfolded and polyubiquitinated protein levels and chymotrypsin‐like proteasome activity in all groups studied.

**Figure 2:**
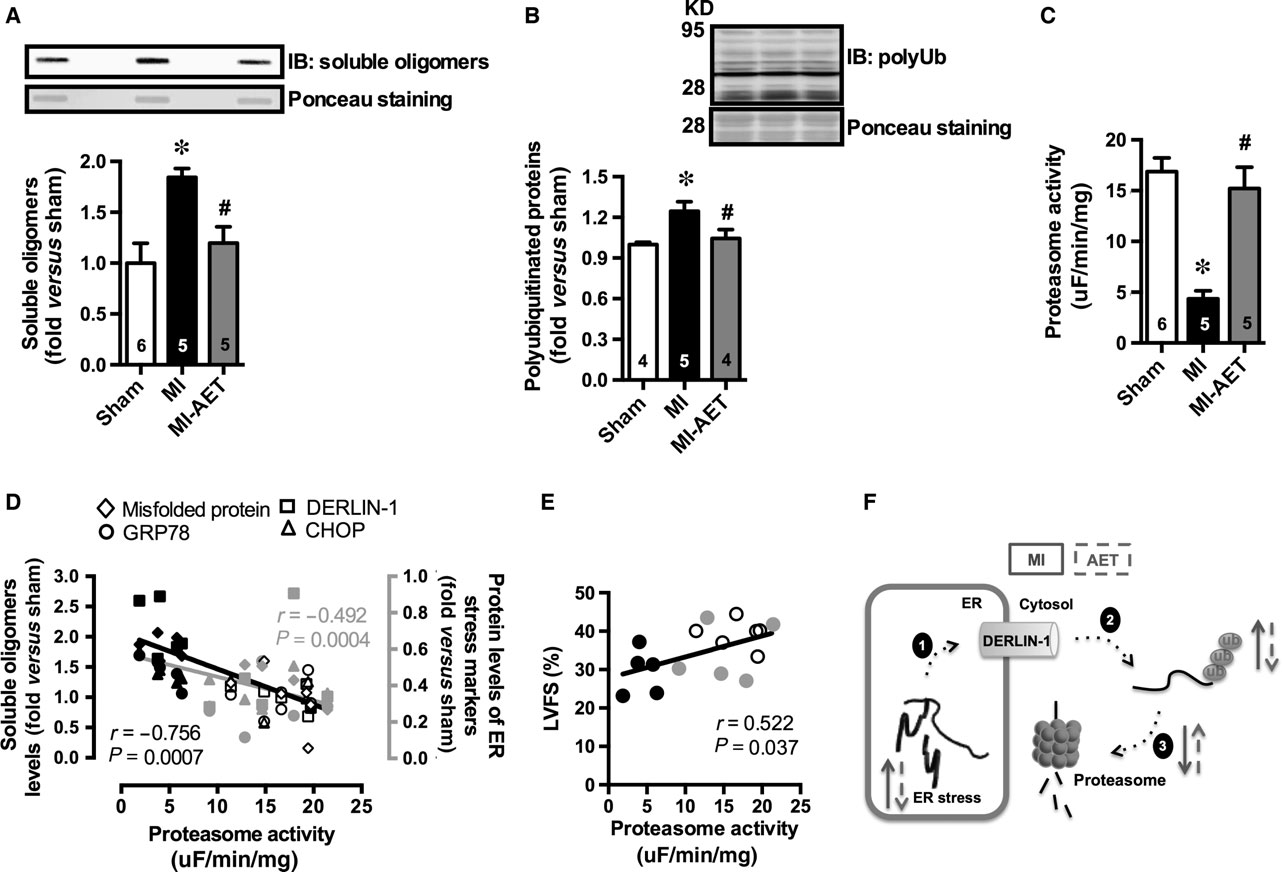
Effect of aerobic exercise training (AET) on misfolded proteins accumulation, levels of polyubiquitinated proteins and proteasome activity in Sham (white), MI (black) and MI under AET (grey). Misfolded protein accumulation evaluated by levels of soluble oligomers (**A**), levels of polyubiquitinated proteins (**B**), chymotrypsin‐like proteasome activity (**C**), correlation between chymotrypsin‐like proteasome activity and protein levels of both misfolded and ER stress markers (GRP78, DERLIN‐1 and CHOP) (**D**), correlation between proteasome activity and cardiac function (**E**), and schematic illustration of the effect of AET on ER protein quality control in post‐MI heart failure rats (**F**). Data presented as mean ± S.E.M. Sham refers to rats submitted to fictitious surgery, MI refers to myocardial infarction and AET refers to aerobic exercise training. * indicates *P* ≤ 0.05 versus sham, ^#^ indicates *P* ≤ 0.05 versus MI. The number of animals in each analysis is shown within the bars.

Hearts of MI rats displayed accumulated cardiac misfolded proteins and increased levels of polyubiquitinated proteins associated with a drastic reduction in chymotrypsin‐like proteasome activity (Fig. 2A–C). These data suggest that MI impairs the cardiac protein quality control through a reduction in proteasome activity, which is known to contribute to ER stress. In fact, we have observed a significant negative correlation between cardiac chymotrypsin‐like proteasome activity and protein levels of both misfolded and ER stress makers (Fig. 2D). Conversely, a positive and significant correlation was observed between cardiac chymotrypsin‐like proteasome activity and cardiac function (Fig. 2E).

Aerobic exercise training restored chymotrypsin‐like proteasome activity (Fig. 2C) in hearts of MI rats paralleled by a reduction in misfolded and polyubiquitinated protein levels (Fig. 2A and B). Altogether, these results indicate that AET by improving cardiac proteasome activity reestablish cardiac protein quality control reducing ER stress, which further attenuates MI‐induced cardiac dysfunction.

## Discussion

Over the last years, several studies have reported that ER stress is involved in the pathophysiology of cardiovascular diseases 14, 15, 16. In fact, ER stress leads to cardiac remodelling and dysfunction 7, and the use of chemical chaperones attenuating ER stress blunts MI‐induced cardiac dysfunction 16. This calls the attention for therapeutic strategies driven to ER stress relief in cardiovascular diseases.

Our study shows, for the first time, that AET‐induced attenuation of cardiac ER stress in post‐MI heart failure rats by recovering cardiac proteasome activity, which is related to improved LV function and exercise capacity. These findings highlight the importance of AET as non‐pharmacological primary therapy for cardiovascular diseases.

Aerobic exercise training‐induced attenuation of LV dysfunction in post‐MI heart failure rats was not followed by a reduction lung oedema. In fact, we cannot exclude that other alterations in cardiac hemodynamic (e.g., diastolic dysfunction, fluid overload) might be less affected by AET or could take longer AET period to reduce lung oedema. It has been demonstrated that improved LV function by AET in HF animals is associated with improved cardiac calcium handling, mitochondrial function and reduced neurohumoural hyperactivity 2, 3, 11. Here, we provide evidence that AET‐induced attenuation of cardiac dysfunction is also related to reestablishment of ER homoeostasis.

Myocardial infarction‐induced reduction in LVFS was associated with increased cardiac levels of GRP78, DERLIN‐1 and CHOP, which are proteins involved in UPR‐coordinated protein folding and degradation, and cell apoptosis respectively 6, 15, 17. However, no changes were observed in VCP levels.

DERLIN‐1 and VCP are misfolded protein transporters from ER lumen to cytosol (DERLIN‐1) and within cytosol (VCP) 15, 17. Both DERLIN‐1 and VCP are involved in ER protein degradation, a process known as ER‐associated protein degradation that comprehends three steps by which misfolded proteins are: translocated from ER to cytosol, ubiquitinated and further degraded by proteasome 17. In our MI model, we showed increased levels of DERLIN‐1, but not VCP. These results suggest that only DERLIN‐1 is responsive to MI‐induced UPR activation, which might be due to its ER location while VCP is a cytosolic protein. In fact, DERLIN‐3, a DERLIN‐1 homologue, drives misfolded protein clearance attenuating ER stress in both mammal cell lineages and neonate cardiac myocyte 15, 18.

Despite increased DERLIN‐1 protein levels in the hearts of MI rats, we observed an impaired cardiac protein quality control through reduced chymotrypsin‐like proteasome activity, recapitulating previous results from our lab in patients and animal models of heart failure 2, 4. These data suggest that increased levels of DERLIN‐1 do not suffice to relieve MI‐induced cardiac ER stress in our model. Accordingly, increased protein level of CHOP in the hearts of MI rats indicates a shift from pro‐survival to pro‐apoptotic UPR signal.

We have previously demonstrated that AET changes several features that can contribute to cardiac ER stress relief in cardiovascular disease 2, 3. We observed that AET attenuates renin angiotensin and sympathetic system hyperactivation, cardiac calcium imbalance and mitochondrial dysfunction in mice and rat models of heart failure 2, 3, 11. Presently, we extended this knowledge to cardiac ER homoeostasis, since AET improved cardiac chymotrypsin‐like proteasome activity reestablishing the clearance of misfolded proteins. This ultimately leads to ER stress relief demonstrated by AET‐induced reduction in levels of proteins considered markers of ER stress.

### Study limitation

This study shows that exercise training reestablishes cardiac protein quality control and attenuates ER stress, which was related to better cardiac function in post‐MI heart failure rats. Although this study does not provide direct evidence to support cause‐effect relationship between ER stress and MI‐induced cardiac deleterious effect, it is already known that attenuation of ER stress blunts MI‐induced cardiac dysfunction and remodelling by modulating apoptosis and fibrosis 16.

Our findings suggest that AET attenuates ER stress through the reestablishment of proteasome activity in post‐MI heart failure rats. However, we cannot exclude that other beneficial effect of AET, such as reduced neurohumoural systems activation, calcium imbalance, and mitochondrial dysfunction, might contribute to ER stress relief 2, 3, 11.

Collectively, our results provide evidence that AET is an available non‐pharmacological therapy to reestablish cardiac protein quality control attenuating ER stress in post‐MI heart failure rats (Fig. 2F). Our data highlight AET as crucial primary therapy counteracting cardiac ER stress in cardiovascular diseases.

## Conflict of interest

The authors declare that there are no conflicts of interest.

## Author contribution

LHMB, NR and PCB conceived and designed research; LHMB, PRJ, VAV and PMD performed experiments; LHMB, NR, UW and PCB analysed data; LHMB, NR, UW and PCR interpreted results of experiments; LHMB and PCB prepared figures; LHMB and PCB drafted manuscript; LHMB, PRJ, NR, UW and PCB edited and revised manuscript; LHMB, PRJ, NR, PMD, UW and PCB approved final version of manuscript.

## Supporting information


**Figure S1.** Protein levels of endoplasmic reticulum stress markers in sham (white) and Sham under AET (black).
**Table S1**. Physiological parameters.
**Table S2.** Echocardiographic parameters.
**Data S1.** Materials and methods.
