# Supplementary material for: Aerobic exercise training rescues cardiac protein quality control and blunts endoplasmic reticulum stress in heart failure rats
Source: J Cell Mol Med. 2016 Jun 16;20(11):2208–12. doi: 10.1111/jcmm.12894 (PMC5082404; doi:10.1111/jcmm.12894)
Supplement: Supplementary file 1 — Figure S1. Protein levels of endoplasmic reticulum stress markers in sham (white) and Sham under AET (black). Table S1. Physiological parameters. Table S2. Echocardiographic parameters. Data S1. Materials and methods. [file JCMM-20-2208-s001.docx]

**Supplemental material for “Aerobic exercise training rescues endoplasmic reticulum protein quality control in heart failure rats”**

Luiz H. M. Bozi, Paulo R. Jannig, Natale Rolim, Vanessa A. Voltarelli, Paulo M. M. Dourado, Ulrik Wisløff, Patricia C. Brum


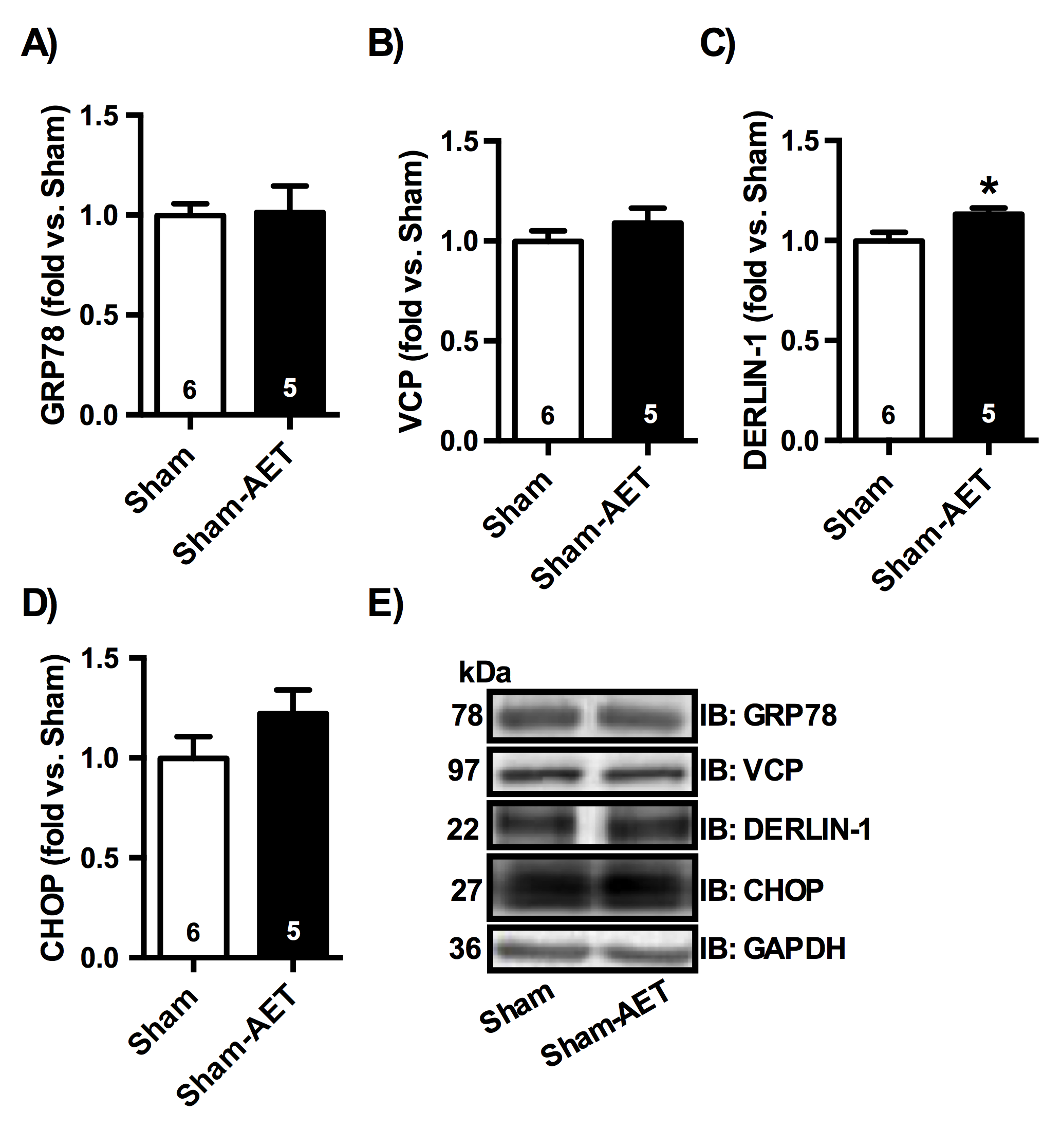


**Figure 1S. Protein levels of endoplasmic reticulum stress markers in sham (white) and Sham under AET (black).** Protein levels of GRP78 **(A)**, VCP **(B)**, DERLIN-1 **(C)** and CHOP **(D)**. Representative immunoblots **(E)**. Data presented as mean ± SEM. Sham refers to rats submitted to fictitious surgery and AET refers to aerobic exercise training. * indicates p ≤ 0.05 *vs.* sham. The number of animals in each analysis is shown within the bars.

**Table S1**. Physiological parameters

| **Parameter** | **Sham (6)** | **MI (5)** | **MI-AET (5)** |
| --- | --- | --- | --- |
| Body weight, g | 408 ± 14.1 | 413 ± 16.7 | 406 ± 6.32 |
| Heart weight, g | 1.12 ± 0.03 | 1.25 ± 0.08 | 1.28 ± 0.03 |
| Heart/body ratio, mg/g | 2.75 ± 0.02 | 3.03 ± 0.08* | 3.15 ± 0.05* |
| Lung wet/dry ratio, g/g | 4.27 ± 0.11 | 4.53 ± 0.07* | 4.56 ± 0.09* |
| MI extension, % | - | 27.6 ± 3.02 | 24.6 ± 5.58 |

Body weight, heart weight, heart/body ratio, lung wet/dry ratio, and myocardial infarction (MI) extension. Data presented as mean ± SEM. * indicates p ≤ 0.05 *vs.* sham. The number of animals used in each analysis is shown within parentheses.

**Table S2.** Echocardiographic parameters

|  | **4 weeks after MI induction** | | |  | **12 weeks after MI induction** | | |
| --- | --- | --- | --- | --- | --- | --- | --- |
| **Parameter** | **Sham (6)** | **MI (5)** | **MI-AET (5)** |  | **Sham (6)** | **MI (5)** | **MI-AET (5)** |
| LVESD, mm | 4.13 ± 0.27 | 5.59 ± 0.55* | 5.42 ± 0.28* |  | 4.55 ± 0.24 | 5.69 ± 0.42 | 5.26 ± 0.54 |
| LVEDD, mm | 7.16 ± 0.13 | 8.17 ± 0.38* | 7.97 ± 0.17* |  | 7.48 ± 0.32 | 8.06 ± 0.45 | 7.96 ± 0.51 |
| HR, bpm | 277 ± 4.54 | 268 ± 8.04 | 264 ± 10.41 |  | 262 ± 7.66 | 267 ± 17.5 | 270 ± 19.5 |

Left ventricular end-systolic diameter (LVESD), left ventricular end-diastolic diameter (LVEDD), and heart rate (HR) under anesthesia of sham, MI and MI-AET groups evaluated 4 and 12 weeks after myocardial infarction (MI) induction. Data presented as mean ± SEM. * indicates p ≤ 0.05 *vs.* sham at the same period of time. The number of animals used in each analysis is shown within parentheses.

**Experimental details**

**Animal model and experimental design**

Male Wistar rats weighting 200-300g were obtained from Medical School, University of São Paulo. They were kept in an animal facility under controlled temperature (21^o^C) with 12:12 hours inverted light:dark cycle, housed five per cage and receiving standard laboratory chow (Nuvital Nutrientes, Brazil) and water *ad libitum*. After a week of acclimatization, rats were anesthetized with ketamine (50 mg/kg ip) and xylazine (10 mg/kg ip), followed by left thoracotomy. The mediastinum was accessed by incision of intercostal muscles between the third and fourth ribs, the heart was carefully exteriorized, and the left anterior descending (LAD) coronary artery was occluded with 6/0 thread. After LAD ligation the thorax was closed, and lung collapse was prevented by rapid withdrawal of air from the pleural cavity. Sham-operated animals underwent similar left thoracotomy and cardiac exteriorization, except for LAD ligation. Four weeks after surgical procedures, rats were submitted to echocardiographic evaluation and treadmill exercise test for assessment of cardiac function and exercise tolerance, respectively. Experimental groups were divided in such a way that all parameters (i.e., exercise tolerance, cardiac structure, and function) were similar among the groups of similar cardiac condition (i.e., sham or MI) (Fig. 1A and B, Table 2S). Sham refers to control group, MI refers to the group of MI rats that remained untrained during the followed 8 weeks of post-infarction surgery, and MI-AET refers the MI rats that underwent to aerobic exercise training (AET) protocol. AET was performed on a motor treadmill, 5 days/week, 60 min/day, for 8 weeks, at 60% of maximal speed that correspond to the maximal lactate steady state workload [1]. Although the optimal exercise intensity to achieve maximal outcome is still a matter of debate, we study the effect of AET at 60% of maximal speed because it is undoubtedly that exercise at moderate intensity is a safety approach to HF patients [2]. At the end of the AET protocol, cardiac function and exercise capacity were re-analysed. A subset of Sham-AET group was compared to Sham group in a separated experiment to test whether AET would change protein levels of ER stress markers independently of MI.

Forty-eight hours after the last exercise training session, all rats were anaesthetized with ketamine (50 mg/kg ip) and xylazine (10 mg/kg ip) and euthanized by decapitation. Heart and lung were carefully removed and weighed (wet weight). To evaluate lung water content, lung was then dried for 72 hours at 60^o^C and re-weighted to verify the lung wet/dry ratio, which was considered an index of lung edema. All procedures were performed in accordance with the *Guide for the Care and Use of Laboratory Animals* (NIH, USA) and were approved by the University of São Paulo’s Ethical Committee (#2011/45).

**Echocardiographic evaluation**

Rats underwent M-Mode echocardiographic examination at forth and twelfth weeks after LAD ligation. Rats were anesthetized with ketamine (50 mg/kg ip) and xylazine (10 mg/kg ip) and were placed in a supine position. Cardiac dimensions were evaluated using an echocardiographer (Acuson Sequoia model 512, Siemens, USA) equipped with a 14-MHz linear transducer. Left ventricular fractional shortening (LVFS) was calculated by the formula: LVFS (%) = [(LVEDD - LVESD)/LVEDD] X 100, where LVEDD means Left Ventricular End-Diastolic Diameter and LVESD means Left Ventricular End-Systolic Diameter. Measurements of echocardiographic examination followed the recommendations of the American Society of Echocardiography [3]. Echocardiography was performed by an experienced researcher blinded to animal´s identity (P.M.D.).

**Graded Treadmill Exercise Test**

To assess the exercise tolerance, rats were submitted to a graded treadmill exercise test at the 4 and 12 weeks after surgeries. Animals were adapted to treadmill exercise during five days (10 minutes each day) before test. The test started at 6 m/min and speed was increased by 3 m/min every 3 minutes until rats were unable to run due to exhaustion [1].

**Myocardial infarction extension**

Heart slices were fixed in 4% buffered formalin, embedded in paraffin for routine histological processing, transversely sectioned in 5 mm-thick sections and stained with picrosirius red for analysis of MI extension. Endocardial and epicardial circumferences of the infarcted tissue and left ventricle were determined using Image J software (NIH, USA). MI extension was calculated as (endocardial + epicardial circumference of infarcted tissue)/(endocardial + epicardial circumference of the left ventricle) and was expressed as a percentage.

**Western blotting**

Glucose-Regulated Protein 78 (GRP78, Abcam, #ab108613), Valosin-containing protein (VCP) (Thermo Scientific, #MA3-004), DERLIN-1 (Sigma-Aldrich, #SAB4200148), C/EBP homologous protein (CHOP, Cell Signaling, #2895) and polyubiquitinated proteins (Biomol, #BML-PW0930) were evaluated by western blotting in total extracts from the left ventricular remote area. Briefly, heart samples were homogenized in RIPA buffer (50mM Tri-HCl, 150mM NaCl, 0,5% sodium, 1% Triton X-100 adjusted to pH 7.4) containing protease inhibitor cocktail (1:100, Sigma-Aldrich, Brazil), and centrifuged for 15 minutes at 12,000 g and 4^o^C. Supernatant was used for the assay. Protein concentration was measured by Bradford assay (Bio-Rad Protein Assay, Bio-Rad, Brazil). Sample homogenates were mixed with 0.8% (w:v) SDS, 200 mM mercaptoethanol, 0.02% (w:v) bromophenol blue and 40% (w:v) glycerol and submitted to SDS-PAGE. Proteins were electrotransferred to nitrocellulose membranes, followed by incubation with bovine serum albumin (5% BSA, w:v) blocking solution. Primary and secondary antibodies were incubated following manufacturer’s instructions. Antibody detection was performed in a digitalizing unit (ChemiDoc, Bio-Rad, USA) after incubation with Pierce ECL Western Blotting Substrate (Thermo Scientific, USA). Quantification analysis of blots was performed with the use of Image J software (NIH, USA). Samples were normalized to relative changes in GAPDH and expressed as fold of sham.

**Slot blotting**

Accumulation of misfolded protein was evaluated in total extracts from the left ventricular remote area by slot blotting. Briefly, samples homogenate (25 μg protein) was slot blotted onto nitrocellulose membrane, followed by incubation with bovine serum albumin (5% BSA, w:v) blocking solution. Anti-soluble oligomer antibody (Biosource International item #AHB0052, USA) that recognizes misfolded proteins by exposition of hydrophobic sites and secondary antibody were incubated following manufacturer’s instructions. Antibody detection was performed in a digitalizing unit (ChemiDoc, Bio-Rad, USA) after incubation with Pierce ECL Western Blotting Substrate (Thermo Scientific, USA). Quantification analysis of blots was performed with the use of Image J software (NIH, USA). Samples were normalized to relative changes in Ponceau staning and expressed as fold of sham.

**Proteasome activity**

Chymotrypsin-like proteasome activity was assayed in the total lysate from ventricular remote area using the fluorogenic peptide Suc-Leu- Leu-Val-Tyr-7-amido-4-methylcoumarin (LLVY-MCA, Enzo Life Sciences item #P802-0005, USA). Assays were carried out in a microtiter plate by diluting 25 mg of cytosolic protein into 200 mL of 10 mM MOPS, pH 7.4 containing 25 mM LLVY-MCA (substrate), 25 mM ATP and 5.0 mM Mg2+. Rate of fluorescent product formation was measured with excitation and emission wavelengths of 350 and 440 nm, respectively.

**Statistical analysis**

Shapiro-Wilk test was used to verify normal distribution of the data. Student’s *t*-test was used to analyze data presented in Figure 1S. All other data were compared among groups by one-way ANOVA. In case of statistical significance, Duncan *post hoc* testing was applied. Pearson’s correlation coefficient was used to detect linearity between variables. All results are presented as mean ± standard error of the mean (SEM) and statistical significance was considered achieved when p ≤ 0.05.

**References in supplementary material**

1. **Ferreira JC, Rolim NP, Bartholomeu JB, et al.** Maximal lactate steady state in running mice: effect of exercise training. *Clin Exp Pharmacol Physiol*. 2007; 34: 760-5.

2. **Ades PA, Keteyian SJ, Balady GJ, et al.** Cardiac rehabilitation exercise and self-care for chronic heart failure. *JACC Heart Fail*. 2013; 1: 540-7.

3. **Picard MH, Adams D, Bierig SM, et al.** American Society of Echocardiography recommendations for quality echocardiography laboratory operations. *J Am Soc Echocardiogr*. 2011; 24: 1-10.
